# Small RNA profiling of Dengue virus-mosquito interactions implicates the PIWI RNA pathway in anti-viral defense

**DOI:** 10.1186/1471-2180-11-45

**Published:** 2011-02-28

**Authors:** Ann M Hess, Abhishek N Prasad, Andrey Ptitsyn, Gregory D Ebel, Ken E Olson, Catalin Barbacioru, Cinna Monighetti, Corey L Campbell

**Affiliations:** 1 Department of Statistics, Colorado State University, Fort Collins, Colorado, 80523, USA; 2 Department of Pathology, University of New Mexico School of Medicine, Albuquerque, NM, 87131, USA; 3 Microbiology, Immunology, and Pathology Dept, Colorado State University, Fort Collins, Colorado, 80523, USA; 4 Arthropod-borne Infectious Diseases Laboratory; Colorado State University, Fort Collins, Colorado, 80523, USA; 5 Life Technologies, Foster City, CA, 94404, USA; 6 Department of Biochemistry and Molecular Biology, Colorado State University, Fort Collins, Colorado, 80523, USA

## Abstract

**Background:**

Small RNA (sRNA) regulatory pathways (SRRPs) are important to anti-viral defence in mosquitoes. To identify critical features of the virus infection process in Dengue serotype 2 (DENV2)-infected *Ae. aegypti*, we deep-sequenced small non-coding RNAs. Triplicate biological replicates were used so that rigorous statistical metrics could be applied.

**Results:**

In addition to virus-derived siRNAs (20-23 nts) previously reported for other arbovirus-infected mosquitoes, we show that PIWI pathway sRNAs (piRNAs) (24-30 nts) and unusually small RNAs (usRNAs) (13-19 nts) are produced in DENV-infected mosquitoes. We demonstrate that a major catalytic enzyme of the siRNA pathway, Argonaute 2 (Ago2), co-migrates with a ~1 megadalton complex in adults prior to bloodfeeding. sRNAs were cloned and sequenced from Ago2 immunoprecipitations. Viral sRNA patterns change over the course of infection. Host sRNAs were mapped to the published aedine transcriptome and subjected to analysis using edgeR (Bioconductor). We found that sRNA profiles are altered early in DENV2 infection, and mRNA targets from mitochondrial, transcription/translation, and transport functional categories are affected. Moreover, small non-coding RNAs (ncRNAs), such as tRNAs, spliceosomal U RNAs, and snoRNAs are highly enriched in DENV-infected samples at 2 and 4 dpi.

**Conclusions:**

These data implicate the PIWI pathway in anti-viral defense. Changes to host sRNA profiles indicate that specific cellular processes are affected during DENV infection, such as mitochondrial function and ncRNA levels. Together, these data provide important progress in understanding the DENV2 infection process in *Ae. aegypti*.

## Background

Small RNA (sRNA) regulatory pathways (SRRPs) control gene expression through a variety of mechanisms [1]. Components of the microRNA, small interfering (siRNA), and PIWI RNA pathways, three major SRRPs, are present in mosquitoes [2]. In each of these pathways, gene expression is regulated in the cleavage and degradation of mRNAs. Cellular processes as diverse as development, anti-viral defense and maintenance of the germline are controlled by these mechanisms [3-6]. In general, the size of the cleavage products reveals the pathway(s) by which degradation occurs [7]. In mosquitoes and other invertebrates, siRNAs of ca. 21-22 nts are expected to be produced by a Dicer-2/R2D2/Argonaute 2 (Ago2) dependent cleavage mechanism, whereas microRNAs (ca. 21-22 nts) are produced by a Dicer-1/Loquacious/Ago1 dependent mechanism [8,9]. Intriguingly, components from these two pathways do not function exclusively from one another. Dicer-2 and an alternate spliceform of Loquacious interact to produce endogenous siRNAs (endo-siRNAs) [10,11]. This alternate pathway is also an important regulator of host gene expression and selfish genetic elements [12]. PIWI pathway products, piRNAs, 24-30 nts in length, are produced in a Dicer-independent manner [13]. Moreover, an additional sRNA size class has been described in the anti-Ago2 antibody immunoprecipitation of unusually small RNAs (usRNAs) (ca. 13-19 nts) [14].

Triggers for SRRPs are only partially understood. The anti-viral and endo-siRNA pathways have a double-stranded RNA trigger which activates processing and loading of an 20-23 nt siRNA guide strand [15]. Once loaded, the RISC may be recycled. The miRNA pathway relies on microRNA-encoding genes that are processed in a DGCR8/Drosha-dependent manner [16]. In contrast to siRNAs, miRNAs, also 20-23 nts, bind to target transcripts with imperfect complementarity. PIWI pathway sRNA biogenesis is less understood but likely involves a single-stranded RNA trigger (reviewed in [7]).

Mosquito-borne dengue virus is a human health threat in tropical urban areas and causes sporadic outbreaks in the southern US [17,18]. It is transmitted to humans by aedine mosquitoes and has bypassed the requirement for an enzootic amplification cycle, thus increasing the threat to public health. Arboviruses must successfully replicate in mosquitoes, escape anti-viral defense, and then invade salivary glands in order to be transmitted during blood feeding to subsequent hosts. Using radioisotopic detection, newly replicated Dengue virus serotype 2 (DENV2) genomes can be detected in *Ae. aegypti *Higg's White Eye (HWE) midguts, the initial site of infection, as early as 4 days post infection (dpi), and viral interfering sRNAs (viRNAs) at 8 dpi [6,19]. The best described anti-viral RNAi pathway relies on a Dicer-2 dependent mechanism whereby the Ago2 endonuclease cleaves target RNAs [20]. Silencing of RNAi component transcripts *Ago2*, *R2D2 *and *Dicer-2 *in *Ae. aegypti *increases DENV2 titers; therefore these components play an important role in controlling arbovirus replication [3,6,21]. Another component of the RNA-induced Silencing Complex (RISC) is Tudor-SN (TSN), a transcriptional co-factor [3,22]. Given the presence of a functional RNAi pathway, it remains a mystery as to how arboviruses overcome anti-viral defense to establish persistent infections and perpetuate the arbovirus disease cycle.

sRNAs represent the product of host mRNA or viral RNA cleavage in an RNAi-specific manner. Detection and characterization of RNAi pathway degradation products in arbovirus-infected mosquitoes lends insight into the interplay between virus and vector at a level of sensitivity not formerly possible. The goals of this study were to a) characterize changes in viRNA production and b) to identify host processes that are differentially regulated by RNAi over the course of infection. DENV2 Jamaica 1409 (JAM1409) was used to infect its natural mosquito vector, *Aedes aegypti*. Most current RNA deep sequencing studies use duplicate technical replicates. By using triplicate biological replicates, deep sequencing and rigorous statistical metrics similar to those used for microarrays, we identify products of RNAi pathway activity that are altered in DENV2-infected mosquitoes. The resulting data provide a basis for determining cellular pathways important to virus infection. This analysis is unique in that we focus on only those gene targets which are cleaved by post-transcriptional SRRPs producing sRNAs from 13-30 nts. Therefore, targets may be revealed that would not be identified using traditional microarray approaches. Alterations to gene expression levels that are controlled at the transcriptional level or by mechanisms of the de-capping or de-adenylation mRNA decay pathways will not be considered here [23].

## Results

### Virus feeding

*Ae. aegypti *Rexville D-Puerto Rico were fed a blood meal containing DENV2 Jamaica 1409 and negative controls were fed blood with an equivalent volume of un-infected insect cell culture homogenate. As with previous studies [24], the mosquitoes had an infection rate of 50% at 9 dpi and geometric mean titers of 2.5 log10 plaque-forming units (pfu) per mosquito.

### RNAi machinery components

**Figure 1:**
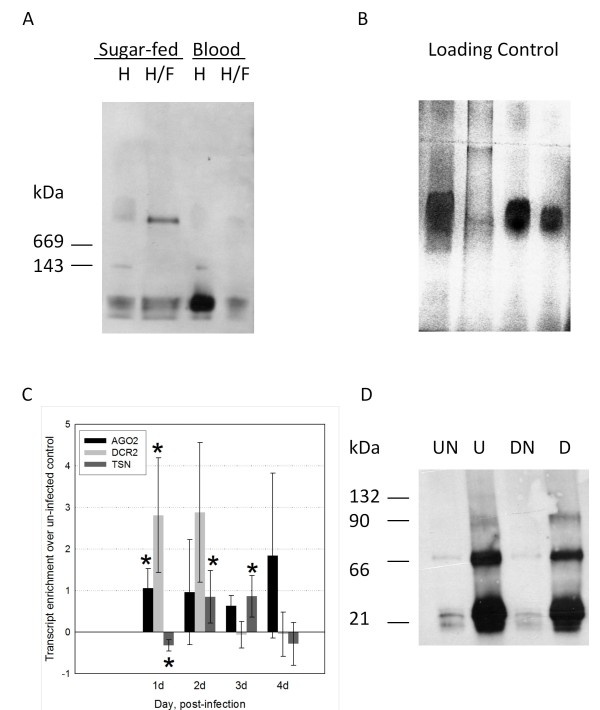
**Antiviral RNAi components are expressed and active in *Ae. aegypti***. A) Ago2 associates with a high MW complex in hemolymph and fat body prior to a blood-feeding. HWE strain hemolymph (collected through proboscis) or hemolymph collected with fat body before and 1 day following a blood meal. About 30 μg protein was separated on a 3-10% Blue Native gel and subjected to immunoblot analysis using anti-Ago2 antibody. 'H', hemolymph, 'H/F', hemolymph with fat body. Size markers show position of proteins of known molecular weight (not shown). B) Silver stained gel shows loading control. C) RNAi component transcripts are modulated during DENV2 infection. Relative changes in DENV2-infected HWE midgut transcript levels detected by qRT-PCR. Significant changes over controls are marked with asterisks (p ≤ 0.05, Mann-Whitney U test); error bars depict standard error of three biological replicates. Pools of 5 midguts were used in each replicate. Relative transcript levels were calculated using the delta-delta Ct method, using ribosomal protein S7 as a reference standard. Enrichment is relative to that of un-infected blood-fed control mosquitoes. D) Western blot of immunoprecipitated products (IP) from pools of 20 DENV2-infected RexD mosquitoes. 'UN', Un-infected blood-fed control mosquitoes collected at 2 dpf (days post-feeding), probed with non-immune serum; 'U', un-infected blood-fed mosquito Ago2 antibody IP; 'DN', Dengue/blood-fed mosquitoes collected at 2 dpi, probed with non-immune serum; 'D', Dengue/blood-fed mosquito Ago2 antibody IP. Size markers show approximate molecular weight of bands shown.

We performed a series of experiments to determine how *Ae. aegypti *RNAi pathway components respond to a blood feeding or DENV2 infection. Hemocytes are critical to mosquito immunity, circulate in the hemolymph and harbor DENV2 particles [24,25]. To give an indication of whether RISC complexes are present in hemolymph before blood feeding, thus supporting the hypothesis that mosquitoes mount an anti-viral response upon infection, soluble fractions were collected using two different methods, separated and probed with anti-Ago2 antibody. High molecular weight complexes containing Ago2 are present in cells from hemolymph/fat body fraction prior to a blood meal and depleted at 1 day post-blood feeding (Figure 1A-1B). Purified hemolymph from sugar-fed and blood-fed females showed a 143 kDa species, and all samples showed the lower molecular bands that are commonly seen in *Ae. aegypti *(Figure 1A, D) [3].

To determine whether *Ago2, Dicer-2 *or *TSN *expression levels are modulated during DENV2 infection, we used quantitative real-time PCR to measure component mRNA levels in midguts at the initial site of infection. *Dicer-2 *and *Ago2 *transcript levels were significantly enriched in DENV2-infected midguts over un-infected blood-fed controls at 1 dpi (Figure 1C). At 2, 3, and 4 dpi, variability in *Ago2 *and *Dicer-2 *transcript levels increases, thereby negating significant differences compared to un-infected controls. By 9 dpi, transcript levels are indistinguishable from those of un-infected controls (data not shown). In contrast, TSN transcriptional co-factor levels were depleted at 1 dpi and enriched at 2 and 3 dpi. Immunoprecipitation (IP) of Ago2 complexes from un-infected blood-fed and DENV2-infected mosquitoes (Figure 1D) and subsequent cloning revealed sRNAs of 12 to 21 nts. The sRNA sequences prepared from the IP-cloning were not among those of the over- or under-represented host sRNAs (data not shown). Multiple bands are present in the immunoblot, and there is little difference in the intensity of Ago2 bands when DENV2-infected and blood-fed controls are compared. A faint Ago2 band at 132 kDa is present in un-infected mosquito IPs and not in DENV2-infected mosquitoes.

### Deep sequencing reveals virus-derived usRNAs, siRNAs, and piRNAs

Pools of twenty mosquitoes from three biological replicates each of virus-infected and un-infected blood fed controls were collected at 2, 4, and 9 dpi, for a total of eighteen libraries. sRNAs up to about 40 nts in length were isolated from total RNA and deep sequenced using sequencing-by-ligation. Library sequences were aligned sequentially to the *Ae. aegypti *published transcriptome, (V.1.2, Vectorbase.org, [26,27] and DENV2 viral genome (Genbank accession number M20558). Using this approach, we identified reads mapping to published mRNAs, small non-coding RNAs, novel mRNAs and the viral genome. Small non-coding RNAs, such as tRNAs and small nuclear RNAs, included in the published aedine transcriptome were also analyzed, because recent evidence indicates that they may be regulated by RNAi-dependent mechanisms [28].

**Figure 2:**
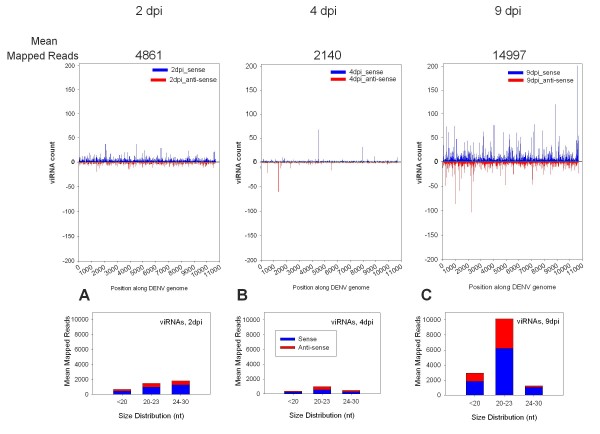
**viRNA profiles change over the course of DENV2 infection**. A) 2 days post-infection (dpi). B) 4 dpi. C) 9 dpi. Top panel shows mean mapped reads and count distribution along DENV2 genome for a representative library at each time point. Bottom panel shows mean viRNA distribution by sRNA size group. Blue and red bars indicate sense and anti-sense viRNAs, respectively.

viRNA reads aligning to the DENV2 JAM1409 genome represented 0.005%- 0.06% of total filtered reads over the course of the infection (Figure 2). Mapped reads included both sense and anti-sense viRNAs, and there was replicate-to-replicate variation in the number of mapped viRNAs (data not shown). sRNAs from un-infected controls aligned to the viral genome indicate the level of false positive matches (Additional File 1A, data not shown). The distribution and abundance of viRNA reads changed over the course of infection. 4861 mean mapped viRNA reads were identified at 2 dpi, 2140 at 4 dpi and ~15,000 at 9 dpi. At 2 dpi, viRNAs represent RNAi-mediated degradation of ingested virus [19]. There were slightly fewer 20-23 nts viRNAs than (37%) than 24-30 nts viRNAs (46%) (Figure 2). At 4 dpi, very few viRNAs were seen. This result was unexpected, because full-length viral genomes have been observed in midguts at this time period [19]. The size distribution among 20-23 nt and 24-30 nt sRNA size groups was 55% and 26%, respectively. By 9 dpi, viRNAs were most abundant and represented about 0.06% of total library reads; 71% and 9% have lengths of 20-23 nts and 24-30 nts, respectively. viRNAs of 20 to 30 nts from a representative library show a slight G/C bias in base composition at the 3' end and a slight bias for 'A's along the length of the sRNA (Additional File 1B). Endo-siRNAs (20-23 nts) from drosophilids show a similar bias [12]. However, sense strand viRNAs of 24-30 nts showed no preference for a 'U' at the 5' end and only a slight bias for 'A' near position 10, as reported elsewhere [29,30]. Although host-derived piRNAs are expected to have a preference for an 'A' at position 10, this feature is not always seen in viRNAs of 24-30 nts [29-31]. We asked whether the lack of a U at the 5' end was an artifact of read alignment by looking at all the bases immediately 5' to the matched read, as well as immediately 3' to the 5' end. We found no preference for a U in either case (data not shown). Further, there is no primer sequence at the 5' end of sRNA sequenced reads in the SOLiD platform. We asked whether the lack of a 5' U could be unique to *Ae. aegypti *by looking at mosquito-derived Sindbis virus viRNAs generated by Illumina sequencing and analyzed using NextGENe software. In this case, a preference for a U at the 5' end of positive sense viRNAs of 24-30 nts was observed (data not shown). Therefore, the lack of a predicted 'U' at the 5' end of viRNAs in the current data set is either unique to DENV infection but not SINV infection or a previously unreported artifact of the Illumina or SOLiD platforms.

### Host sRNA Profiles

To identify host factors that are differentially regulated by SRRPs during DENV2 infection, we asked whether sRNA profiles mapping to host RNAs change in DENV2-infected mosquitoes compared to un-infected controls. sRNA profiles were categorized by the target RNA to which they mapped, as well as by sRNA size group. Changes to ncRNAs were also measured, because recent evidence suggests that they are regulated by RNAi pathway activity [28,32]. For this line of inquiry, we did not distinguish between 20-23 nt siRNAs, endo-siRNAs, or microRNAs. We reason that enriched sRNA profiles for a given target represent the product of enhanced target cleavage, in the absence of concomitant transcriptional repression or mRNA decay [28,33]. Conversely, depleted sRNA profiles among the DENV2-infected pools would be indicative of fewer degraded mRNAs. We defined a single sRNA profile as the number of reads mapped to a single target transcript. The presence of sRNAs aligning to a given transcript would be expected to change sporadically across the three biological replicates if they arose through non-specific decay events. Moreover, non-specific decay events should produce sRNAs across all size groups in similar frequency. Therefore, we expect that sRNA levels with statistically significant enrichment or depletion represent altered RNAi pathway activity.

**Figure 3:**
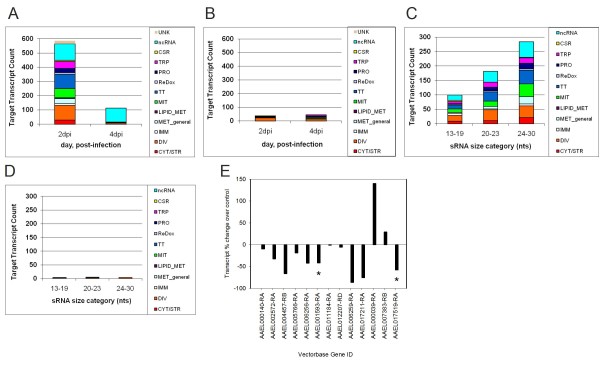
**sRNAs mapped to host target RNAs are enriched or depleted during DENV2 infection and show an inverse relationship to transcript levels**. A) Enriched sRNAs categorized by target functional group in DENV2-infected samples over un-infected blood-fed controls. B) Depleted sRNAs categorized by target functional group in DENV2-infected samples over controls. C) Enriched sRNAs at 2 dpi categorized by sRNA size group. Targets of unknown function are not shown. D) Depleted sRNAs at 2 dpi categorized by sRNA size group. Targets of unknown function are not shown. 'ncRNA', non-coding RNAs, 'CSR', chemo-sensory receptor, 'TRP', transport (signal transduction, ion transport, transmembrane transport), 'PRO', protease, 'ReDox', oxidative reductive components not associated with the mitochondria, 'TT', Transcription/Translation mRNAs, 'MIT' mitochondrial function, 'LIPID_MET' Lipid_Metabolism, 'MET', general metabolism, 'IMM', immunity, 'DIV', diverse function, 'CYT/STR', cytoskeletal/structural. E) Selected target mRNAs were subjected to qRT-PCR analysis in pooled midguts. Bars represent percent change in 2 dpi DENV-2 infected RexD *Ae. aegypti *midguts versus un-infected control midguts from the same time-point. The Delta-delta Ct analytical method was applied and ribosomal protein S7 was used as reference standard. Target transcripts not maintaining the expected inverse relationship with sRNA profiles are marked with an asterisk.

The RNA-seq program edgeR was used determine the significance of sRNA profile enrichment or depletion for sense and anti-sense sRNAs across all three replicates for each timepoint [34]. Sense strand reads were more abundant than anti-sense reads. All target transcripts are categorized by read orientation in Additional File 2. A cut-off value of 0.05 False Discovery Rate (FDR) was used to determine whether changes were statistically significant [35]. sRNA populations mapping to mRNAs and ncRNAs were grouped into functionally similar categories. Figure 3 shows functional categories for which sRNA profiles were modulated over the course of infection. At 2 dpi, 555 unique targets showed enriched sRNA profiles compared to controls, whereas at 4 dpi, only 67 targets had significantly enriched sRNA profiles (Figure 3A and Additional File 2). Under-represented sRNA profiles were much less abundant; 43 unique targets showed depleted sRNAs in DENV2-infected mosquitoes at 2 dpi, and 44 targets showed depleted sRNAs at 4 dpi (Figure 3B). Very few differentially abundant profiles were observed at 9 dpi, therefore they were excluded from further analysis (Additional File 2).

2 dpi sRNA profiles presented in Figures 3A and 3B were distributed by sRNA size group and presented in Figures 3C and 3D. sRNAs were required to maintain statistically significant enrichment (Figure 3C) or depletion (Figure 3D) within their particular size group. At 2 dpi, sRNAs mapped to targets of mitochondrial function (MIT), transcription and translation (TT), as well as ncRNAs, i.e. tRNAs and U RNAs, are the most abundant of all sRNAs in the 24-30 nt size range (Figure 3C). The sRNAs from Figure 3C were analyzed to determine whether 12-19 nt usRNAs, 20-23 nt sRNAs, or 24-30 nt piRNAs might be modulated simultaneously for the same target. Additional File 3 depicts the number of targets that share multiple sRNA size classes at 2 and 4 dpi.

Quantitative RT-PCR was used on an independent biological replicate to test our hypothesis that sRNA profiles of host genes would be inversely proportional to mRNA levels, and thus are indicators of RNAi-dependent mRNA degradation. Most changes to gene expression at the early timepoints should occur in infected midguts. Eleven of thirteen selected RNA targets, sampled at 2 dpi, showed the expected inverse relationship at the timepoint at which sRNA profiles changes were observed (Figure 3E).

## Discussion

We used deep sequencing of multiple biological replicates to characterize DENV2-derived viRNAs. We showed that the pattern of viRNA production changes dramatically over the course of infection and that a functional RNAi pathway is not sufficient to clear DENV2 infection in *Ae. aegypti*. The presence of sense and anti-sense viRNAs at 2 dpi indicates that DENV2 has initiated replication by this time period and the nascent viral genomes are accessible to RNAi pathway activity. However, by 4 dpi, mean mapped reads have dropped by half. Because a previous study showed evidence of full-length viral genomes at 4 dpi, we speculate that viral genomes are protected from RNAi-mediated degradation [6]. This time period also marks the prelude to expanded virus infection in the midgut prior to dissemination and therefore could be a critical window wherein the vector competence phenotype is determined for a given individual. Moreover, early host responses may determine whether a persistent virus infection will be established in susceptible mosquitoes or, alternatively, cleared in resistant individuals. Our host sRNA profile data support this hypothesis. Significant differences in sRNA profiles across mosquito pools are most pronounced at 2 dpi, lessened at 4 dpi and not detectable by 9 dpi. This could be due to increasingly individualized host responses as the infection progresses.

This is the first demonstration that viRNAs of 24-30 nts are a product of arbovirus infection using a natural vector/virus combination and important supportive evidence that the piRNA pathway plays a role in anti-viral defense in mosquitoes, as has been postulated previously [21,31]. viRNAs are most abundant in the 24-30 nt size group at 2 dpi. As infection progresses, the viRNA size range is altered, until at 9 dpi, the predominant population of viRNAs are from 20-23 nts, indicative of a dominant Dicer2-dependent RNAi response.

We show that high molecular weight complexes containing Ago2 are present in cells of the mosquito's open circulatory system prior to infection. This is the first evidence from mosquitoes showing the presence of these high molecular weight complexes. Multiple anti-Ago2 antibody cross-reacting bands are present in whole mosquitoes, suggesting that several Ago2 isoforms are present [3]. The 116 kDa Ago2 protein previously identified in mosquito midguts was not seen in IPs of whole mosquitoes [3], likely because of preferential binding of smaller molecular weight products. Moreover, a 66 kDa alternate spliceform has been identified and could be represented in the 66 kDa IP band (data not shown, CLC). We also immunoprecipitated 20-21 nt sRNAs and usRNAs (13-19 nts) from aedine mosquitoes using anti-Ago2 antibody. The presence of the usRNA size class adds to the complexity of possible regulatory control mediated by Ago2. Gene expression of anti-viral RNAi components is enhanced early in DENV2 infection, in contrast to alphavirus infection, which does not produce significant alteration to either *Ago2 *or *Dicer-2 *transcript levels [3].

Total transcriptome-mapped reads grouped by sRNA size group show an overall increase in 24-30 nt size group in DENV-infected libraries (Additional File 1C); although this result is not statistically significant, a similar result was also observed in West Nile Virus-infected *Culex pipiens quinquefasciatus *(data not shown). Virus-derived usRNAs were also observed, however it is unclear the role these sRNAs play in the infection process.

Host factor analysis relied on statistically significant differences in sRNA profiles of DENV2-infected mosquitoes across three biological replicates. sRNAs were mapped unambiguously to target mRNAs on the published aedine transcriptome. If mapped sRNAs were the result of mRNA decay by RNAi-independent mechanisms, we would expect their profiles to change sporadically across the independent replicates and thus be removed during statistical analysis.

sRNA count data for each target was compared between DENV2-infected pools and those of blood-fed controls. Changes to host sRNA profiles were observed at 2 and 4 dpi but not at 9 dpi. Analysis of target functional groups indicates that mRNAs coding for transcription/translation, transport, cytoskeletal or structural components, and mitochondrial functional processes, especially oxidative phosphorylation and oxidation/reduction are differentially degraded by RNAi pathways during DENV2 infection. These processes have all been previously identified as being important to flavivirus entry, replication and dissemination [36-39]. Viruses must usurp canonical host pathways in order to replicate and establish persistent infections in host mosquitoes. Therefore, these gene expression changes could represent a generalized stress response, bonafide host anti-viral responses or virus manipulation of host processes to facilitate infection. Although further study will be required to tease apart these subtle differences, our data demonstrates that SRRPs are altered early during the course of DENV2 infection.

**Figure 4:**
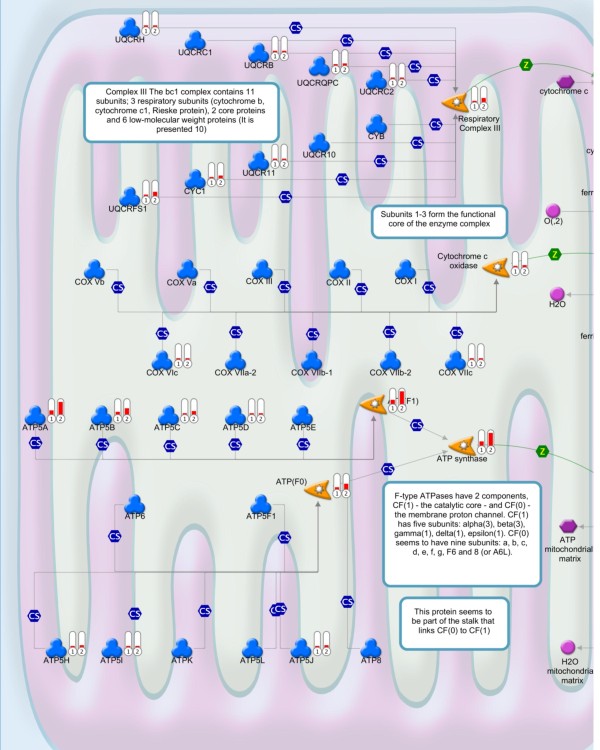
**Predicted alterations in oxidative phosphorylation pathway components in DENV2-infected mosquitoes at 2 dpi**. Differences in sRNA profiles were compared for un-infected controls and DENV2-infected mosquitoes at 2 dpi. Over-represented sRNA targets are revealed in the oxidative phosphorylation pathway, particularly among components of respiratory complex III. Direction and relative scale of sRNA counts for a given target are marked by red bar indicators near the corresponding target genes. Bar 1 indicates un-infected controls; Bar 2 indicates DENV2-infected pools. The legend to GeneGo Metacore pathway maps is given in Additional File 4.

Mitochondrial targets were among the functional groups significantly affected in 2 dpi DENV2-infected samples. The 20-23 nt sRNA size class was the most common size class acting on mitochondrial target mRNAs. Targets involved in ATP production and other aspects of oxidative phosphorylation were especially affected. Key targets are located in respiratory complexes I and III (Figure 4, additional file 4 and data not shown). Similar targets have also been identified in human cells infected with DENV2 [40]. The modulation of mitochondrial targets in DENV2-infected mosquitoes suggests that mitochondria may be stressed during infection, and the host is regulating gene expression to respond to this stress. DENV2 infections are characterized by membrane proliferation in both mammalian and mosquito cells; these membranes are derived from the endoplasmic reticulum [41-44]. Perhaps mitochondrial stress stems from the increased energy load required to re-organize intracellular membranes and support DENV2 infection.

Small non-coding RNAs (ncRNAs), such as tRNAs and small nucleolar RNAs (snoRNAs), are cleaved by Dicer-dependent mechanisms [28,32]. Changes to tRNA and other ncRNA levels could be one mechanism used by hosts in anti-viral defense to slow viral replication. This is supported by the observation that codon usage bias differs among mosquitoes and flaviviruses [45]. Distinct subsets of tRNA and U spliceosomal ncRNAs are affected during DENV infection (Additional File 2). Further study is needed to determine the mechanisms by which ncRNA pattern changes would affect DENV replication.

## Conclusions

Together, these data indicate that profound changes occur in mosquito metabolic pathways early in the DENV2-infection process. Mosquitoes use SRRPs in multiple lines of defense against arboviruses but remain unable to prevent persistent infections. The important features of the DENV2-infection process described here provide a context for future studies to define cell autonomous host responses to arbovirus infection in vector mosquitoes.

## Methods

### Mosquito Infections/Virus stocks

Colonized *Ae. aegypti*, Puerto Rico Rexville D or HWE strains, were reared under standard conditions at 28°C, 80% relative humidity, with a photoperiod of 14:10 (L:D). HWE is a white eye genetic variant of the RexD strain. Adults were provided with a sugar source and water and held in the same conditions during the virus infection extrinsic incubation period.

High passage Dengue serotype 2 Jamaica 1409 (DENV) cultures were prepared by infecting C6/36 *Ae. albopictus *cell culture at an MOI of 0.01 and incubating for 12 days at 28°C at 5% CO_2 _in Minimal Eagles medium. RexD mosquitoes at 4-7 days of age were fed a blood meal containing a 1:1 dilution of DENV in C6/36 cell culture medium and defibrinated sheep blood. Samples harvested at days indicated. Un-infected controls were fed blood diluted 1:1 with C6/36 cell culture medium. Three biological replicates were performed for deep sequencing libraries. DENV2-blood meal titers ranged from 6.7 to 7.8 log plaque forming units (pfu) per ml. Whole mosquito pools were stored in Trizol reagent (Invitrogen) at -80°C. Ten mosquitoes were titered individually using standard methods [3].

### Libraries and Sequencing

Total RNA was extracted from each RexD pool using Trizol (Invitrogen). Small RNAs were isolated from the total RNA using the FLASHPAGE system (Applied Biosystems) and the manufacturer's recommendations. Individual sequencing libraries were prepared using the Applied Biosystem's Small RNA Expression kit. Use of bar-coded primers allowed library pools to be sequenced simultaneously on two slides. 18 libraries were sequenced by ligation based method using the SOLiD system (Applied Biosystems, Foster City, CA). Total reads per library ranged from about 12,000,000 to 49,000,000. Library construction included sRNA purification by size and required a free 5' monophosphate and 3' hydroxyl to allow ligation of adapters, therefore excluding capped mRNAs from library amplification.

### Sequence Analysis

The sequence analysis program NEXTGENe program (SoftGenetics, LLC) version 1.94 or 2.0 was used to align sRNAs in csfasta format to reference genomes in the following order: *Ae. aegypti *transcriptome (AaegL1.2.fa.gz), masked Supercontigs (Liverpool.AaegL1.fa.gz), unmasked contigs (Liverpool.AaegL1.fa.gz), and dengue genome. NEXTGENe uses a proprietary alignment method. The unambiguous alignment setting maps reads to the first perfect match in cases where more than site occurs in the reference sequence. Up to 10% mismatched nts were allowed, to allow for strain-to-strain differences in coding sequences between the RexD strain and the model Liverpool strain. Stringent analytical methods were applied to discover sRNA profile changes that are consistent across biological replicates. The following parameters were used for mosquito transcriptome mapping: Transcriptome alignment, Matching Base Number > = 12, Matching Base Percentage > = 50.0, Alignment Memory Ratio: 1.0, ambiguous mapping: FALSE, Mutation Percentage < = 10.00. "Allsample" output files and Expression Reports were used for data analysis. For viral genome mapping, 5% mutation was allowed, and all other settings were identical.

Relative levels of sRNAs for a given target transcript or segment were calculated in the following way. Only those target transcripts which had an absolute sRNA read count of >10 were used in the analysis. The R module edgeR was used to determine significant changes to sRNA profiles [34]. edgeR relies on an overdispered Poisson model which moderates the dispersion approach with Bayes methods. We used the segment-wise dispersion method with prior.n = 10. A False discovery rate cutoff of 0.05 was used to determine whether a given target mRNA showed significant enrichment or depletion of mapped sRNAs.

Statistical analysis was done in R using Bioconductor [46]. Mapped reads from NextGENe were sorted by sRNA size group (≤ 19, 20-23, 24-30 nts) and orientation. A summary of the distribution of mapped reads by library, orientation and size is given in Additional File 2. Prior to statistical analysis, two levels of filtering were done. First, segments with fewer than 10 reads total across all libraries were dropped from further analysis. In addition, to reduce false positives due to a single outlier, segments where a single library/rep accounted for 70% or more of the total reads were removed from further analysis (ie. a segment with a total of 100 reads with 80 reads coming from a single library would be flagged). Filtering was done separately for each comparison group (ie. orientation and size group). The numbers of segments remaining after filtering as well as the number of segments removed due to a single outlier are given in Additional File 2.

After filtering, comparisons of DENV vs BF were carried out by time point (2, 4 and 9 days post-infection), orientation (forward and reverse) and size group (≤ 19, 20-23 and 24-30). Normalization and testing used edgeR; estimated log2 fold change (logFC) values and p-values were calculated by segment [34,47,48]. edgeR is a Bioconductor software package for examining differential expression of replicated count data. Briefly, an overdispersed Poisson model is used to account for variability and empirical Bayes methods are used to moderate the degree of overdispersion across transcripts. A "segment-wise" dispersion approach (with n.prior = 10) was used. The exact test was used to test for a difference between DENV vs BF. The Benjamini-Hochberg method was used to adjust for multiple testing and control the false discovery rate (FDR) at 0.05 [35]. Gene annotation data was downloaded from Biomart (Biomart.org) [49] and AegyXcel http://exon.niaid.nih.gov/transcriptome.html#aegyxcel. Annotation of transcripts in redundant functional groups relied on the following priorities for functional assignments: 'mitochondrial' functional group included all transcripts that ultimately pertain to mitochondrial function, are located in mitochondrial compartments. This category could include targets that function in transport, transcription, translation, or oxidation/reduction processes. Targets in the 'ReDox' category do not include mitochondrial components.

### Biological Pathway analysis

Enriched or depleted host sRNA profiles listed in Additional File 2 were subjected to pathways analysis using the shadow lists of nearest *Drosophila melanogaster *homologues of *Aedes aegypti *genes. In case of most evolutionally conserved mitochondrial genes, we used shadow lists of human nearest homologue genes admissible as input for pathway analysis software. For preliminary analysis and plots of gene interaction graphs, DroID was used [50]. Oxidative phosphorylation maps were generated using GeneGo Metacore pathway analysis software (GeneGo Inc., St. Josef, MI).

### qRT-PCR

Experimental and analytical methods are similar to those used previously, and primers used for RNAi component PCR were described in a previous report [3]. RNA was extracted from 10 *Aedes aegypti *RexD strain midguts per experimental and control group homogenized in 300 μL TRIzol^® ^(Invitrogen), as per a slightly modified version of the manufacturer's suggested protocol. Isolated RNA re-suspended in 50 μL nuclease-free sterile water and immediately quantified via Nanodrop (Thermo Scientific). Total RNA was aliquoted into 5 ng/μL working solutions and immediately frozen at -80°C until use for qRT-PCR analysis. Primers (Additional File 2) were designed using IDT DNA's online primer design software for qPCR http://www.idtdna.com/Scitools/Applications/RealTimePCR/ and annealed to regions spanning exon-exon junctions. SYBR Green chemistry qRT-PCR was performed using *Power *SYBR^® ^Green RNA-to-C_T_^™ ^1-Step kits(Applied Biosystems) in 20 μL reactions using manufacturer's suggested reagent ratios and 10 ng total RNA per reaction. All gene targets, including the internal housekeeping control gene (RPS7) were screened in triplicate reactions. qRT-PCR was performed on an SDS 7000 machine (Applied Biosystems), and results collected and analyzed using the accompanying SDS 7000 software. Relative measure of differential gene expression was calculated using the ∆∆C_T _method of approximation.

### Immunoprecipitations

Anti-Ago2 antibody (Ab) previously described [3], was used to immunoprecipitate sRNAs from pools of 20 DENV-infected or blood-fed RexD mosquitoes at 2 dpi, using the methods similar to those of Maniataki [51]. Briefly, 5 micrograms anti-Ago2 Ab or non-immune sera were bound to Dyna-beads (Invitrogen) for 45 minutes. Mosquitoes were homogenized in Lysis buffer (20 mM Tris-Cl, 200 mM NaCl, 2.5 mM magnesium chloride, 0.05% NP-40, and 2× EDTA-free Protease inhibitors (Pierce)), an incubated overnight at 4°C on a rocking platform. Immunoprecipitates were rinsed 5 times in Lysis buffer, then extracted with phenol chloroform using the methods of Maniataki. The Applied Biosystems SOLiD sRNA Extraction Kit (Life Technologies) was used to clone small RNAs, and they were sequenced individually using standard methods. sRNA sequence data was obtained for 23 clones using this method.

Immunoprecipitates were also subjected to electrophoresis and western blotting. In this case, immunoprecipitates were diluted in SDS-PAGE buffer and separated on a 4-15% gradient PAGE gel using standard separation methods. Proteins were transferred to PVDF and probed with anti-AGO2 antibody to show the relative size of immunoprecipitated products. Bands on an identical gel containing separated immunoprecipitates were below the detection limit of silver stain detection (data not shown).

### Blue Native PAGE gel

High molecular weight Ago2 complexes were purified from HWE mosquito hemolymph collected with or without fatbody. Hemolymph without fat body was collected by severing the mosquito proboscis and collecting the clear hemolymph released into the tip of a 10 ul pipette, whereas, hemolymph with fatbody was collected from hemolymph released from the hemocoel upon separation of the abdomen and thorax. In either case, the samples were flash-frozen in dry ice and stored at -80°C in 50 mM imidazole/HCl, 50 mM sodium chloride, 2 mM aminohexanoic acid, 1 mM EDTA. Blue Native (BN) gel methods of Wittig et al were used [52]. Prior to BN PAGE separation, samples were spun for 20 minutes at 20,000 × g and 10 ul of 50% glycerol was added to the supernatants. About 30 ug protein for each sample was separated on a 3-10% acrylamide gradient gel prepared in 25 mM imidazole and 0.5 M 6-aminohexanoic acid. The cathode buffer contained 50 mM tricine, 7.5 mM imidazole, 0.02% Coomassie blue G-250, and the anode buffer contained 25 mM imidazole. Proteins were separated at 12 milli-amps for 2 hours in 4°C.

### Immunoblot analyses

PAGE separated proteins were transferred to PVDF using tank transfer at 350 milliamps for 1 hour, blocked with 5% milk for one hour and probed with anti-Ago2 Ab diluted 1:100 [3]. ECL Plus chemiluminescence detection was used, and the blot was exposed to ECL film (Amersham).

## Authors' contributions

CLC conceived the study and performed experiments and analysis. AH did statistical and data analyses. AP contributed pathways analysis. GDE, KEO, and CLC contributed to the manuscript. CLC and AH wrote the paper. ANP performed qRT-PCR. CM and CB performed sequencing and data analyses, respectively. All the authors have read and approved the final manuscript.

## Supplementary Material

Additional file 1 **Additional viRNA profiles**. A. sRNA reads from representative libraries of un-infected controls show non-specific alignment to the DENV2 genome. Panels from left to right indicate, 2, 4, and 9 dpi, respectively. Top panel shows count distribution along DENV2 genome for a representative library at each timepoint. Bottom panel shows mean sRNA distribution by size. Blue and red bars indicate sense and anti-sense sRNAs, respectively. B. viRNA WebLogos. viRNAs from a representative 9 dpi DENV2-infected cohort were separated by size group and subjected to WebLogo sequence alignment http://weblogo.berkeley.edu/ to identify the relative nucleotide frequency at each position. About 20,000 reads were analyzed for the combined categories. C. 24-30 nt piRNAs are more abundant in DENV2-infected samples. Total mean transcriptome-mapped reads of un-infected and DENV2-infected libraries categorized by sRNA size group. Blue and red bars indicate sense and anti-sense viRNAs, respectively. Click here for file

Additional file 2 **Host sRNA Profile Summary Tables**. Summary data categorized by mapped read orientation and sRNA size group. 'Summary' page shows total sRNA reads in pooled libraries for each condition tested. ''Transcripts' shows the number of targets remaining after removing low-abundance (<10 reads) and flagged candidates. "Flagged" segments are those for which a replicate accounted for 70% or more of the total reads; these were deleted from the final analysis. 'Enriched' and 'Depleted' indicate the number of targets showing significant changes in DENV2-infected pools over controls. Significance was determined using the edgeR exact test, and a Benjamini-Hochberg cut-off of 0.05 was used to adjust for multiple testing and control the false discovery rate. The following pages list raw sRNA count data for each target transcript at 2, 4, or 9 dpi. 'DayX sense' shows differential enrichment data for host sense strand sRNAs across all libraries collected at X dpi. Other pages show similar sRNA profiles for anti-sense and sense strand sRNA reads at the indicated collection time. 'Category', indicates target functional category described in Figure 3 legend. 'logFC', log2 fold change in DENV-infected versus control for all sRNAs; 'F_pval', p value of exact test, 'F_FDR', FDR for summed sRNAs. Day2 ncRNA Table shows unique tRNAs represented in the enriched sRNA profiles at 2 and 4 dpi. qRT-PCR Primers Table shows primers used in analysis shown in Figure 3F. Click here for file

Additional file 3 **Targets sharing sRNAs from different size categories**. Venn diagram shows the number of targets that share sRNAs of different size groups for 2 and 4 dpi. Click here for file

Additional file 4 **GeneGo Metacore pathway legend**. Symbols denote objects shown in pathways analysis in Figure 4. Click here for file
