# Supplementary figures and images for: Small RNA profiling of Dengue virus-mosquito interactions implicates the PIWI RNA pathway in anti-viral defense
Source: BMC Microbiol. 2011 Feb 28;11:45. doi: 10.1186/1471-2180-11-45 (PMC3060848; doi:10.1186/1471-2180-11-45)

## Additional File 1A

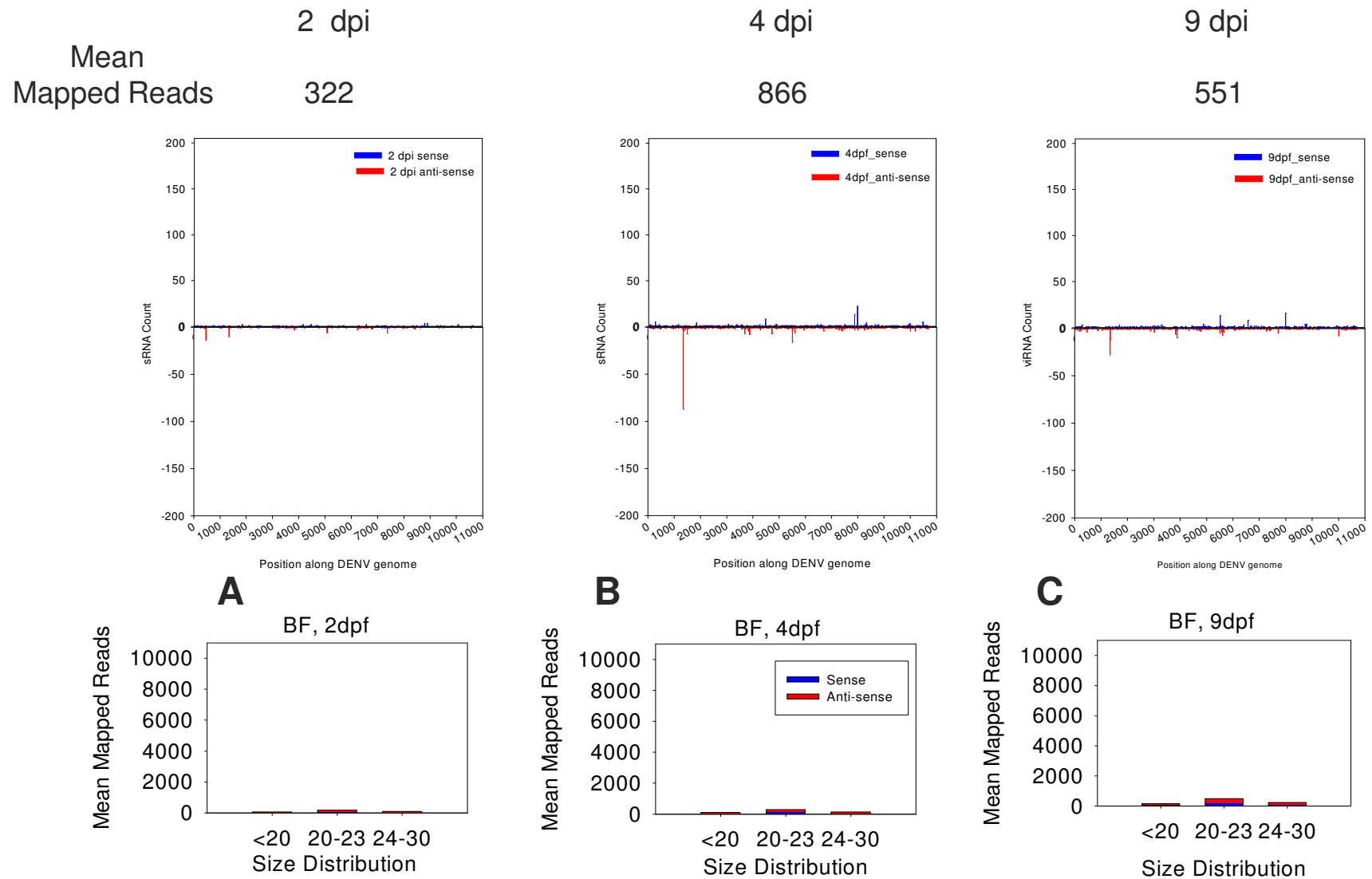

## Additional File 1B

**20-23 nts**

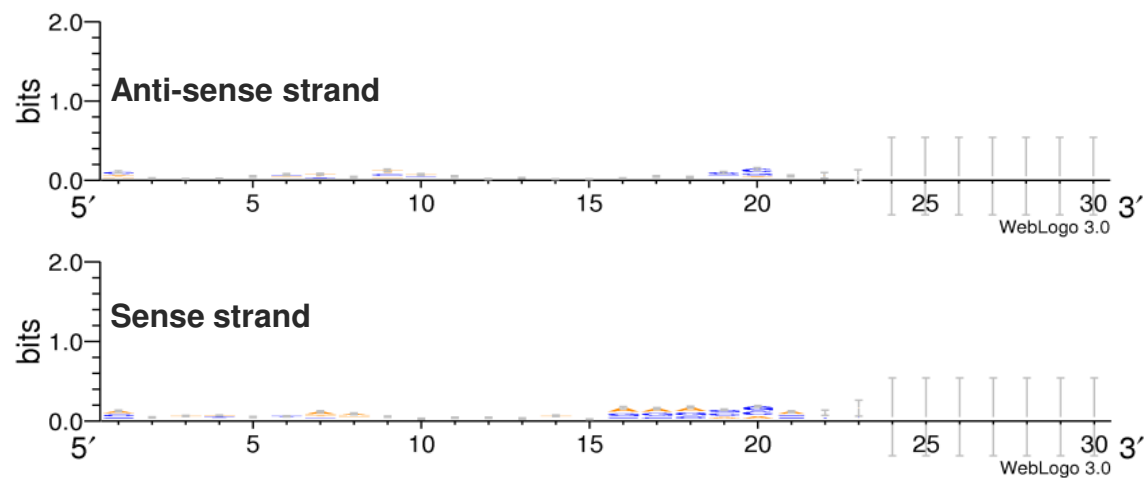

**24-30 nts**

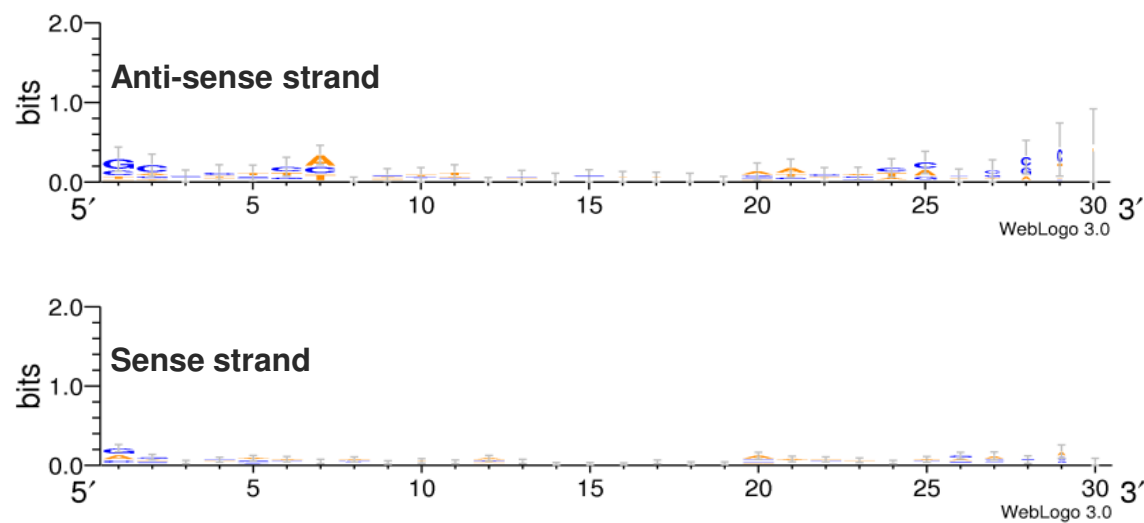

## Additional File 1C

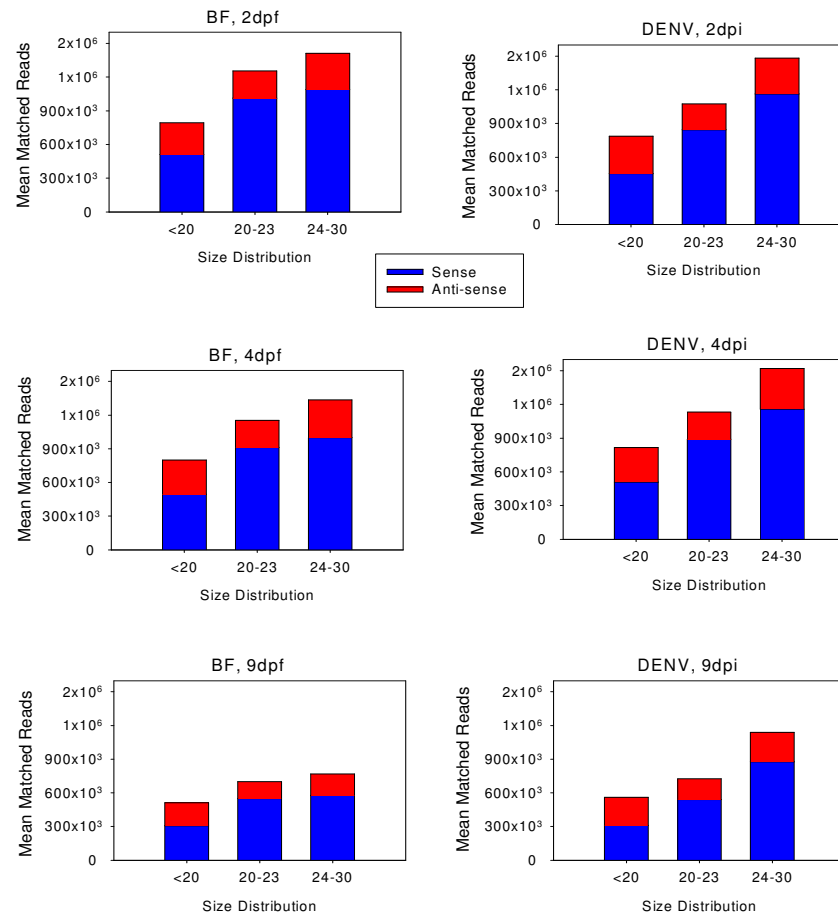

Supplement: Additional file 1 — Additional viRNA profiles. A. sRNA reads from representative libraries of un-infected controls show non-specific alignment to the DENV2 genome. Panels from left to right indicate, 2, 4, and 9 dpi, respectively. Top panel shows count distribution along DENV2 genome for a representative library at each timepoint. Bottom panel shows mean sRNA distribution by size. Blue and red bars indicate sense and anti-sense sRNAs, respectively. B. viRNA WebLogos. viRNAs from a representative 9 dpi DENV2-infected cohort were separated by size group and subjected to WebLogo sequence alignment http://weblogo.berkeley.edu/ to identify the relative nucleotide frequency at each position. About 20,000 reads were analyzed for the combined categories. C. 24-30 nt piRNAs are more abundant in DENV2-infected samples. Total mean transcriptome-mapped reads of un-infected and DENV2-infected libraries categorized by sRNA size group. Blue and red bars indicate sense and anti-sense viRNAs, respectively. [file 1471-2180-11-45-S1.PDF]

## Slide 1
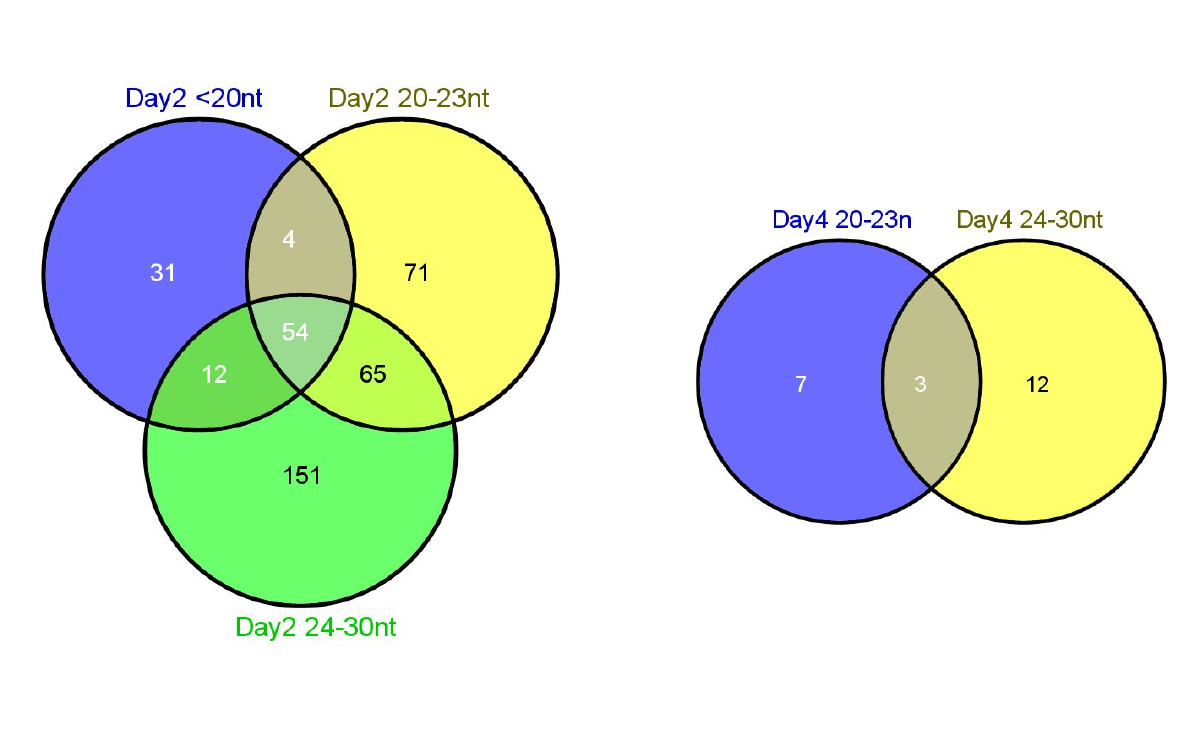

Supplement: Additional file 3 — Targets sharing sRNAs from different size categories. Venn diagram shows the number of targets that share sRNAs of different size groups for 2 and 4 dpi. [file 1471-2180-11-45-S3.PPT]
